# Freeze-Drying in Sucrose Followed by Cryomilling Enables the Formulation of sa-mRNA–LNP Powders for Inhalation

**DOI:** 10.3390/pharmaceutics18010121

**Published:** 2026-01-18

**Authors:** E. M. Jansen, M. J. R. Ruigrok, M. S. Suh, P. M. Ruppel, Xiaole Cui, L. Opsomer, N. N. Sanders, H. W. Frijlink, W. L. J. Hinrichs

**Affiliations:** 1 Department of Pharmaceutical Technology and Biopharmacy, Groningen Research Institute of Pharmacy, Faculty of Science and Engineering, University of Groningen, 9713 AV Groningen, The Netherlands; 2 Laboratory of Gene Therapy, Department of Veterinary and Biosciences, Faculty of Veterinary Medicine, Ghent University, B-9820 Merelbeke, Belgium; 3 Cancer Research Institute (CRIG), Ghent University, B-9000 Ghent, Belgium

**Keywords:** sa-mRNA–LNPs, lyophilization, freezing, freeze-drying, cryomilling, sucrose, stabilization, inhalation, Cyclops DPI

## Abstract

**Background:** Self-amplifying mRNA (sa-mRNA) represents a promising platform for vaccines and gene therapies, offering sustained protein expression at low doses through self-replication. For vaccines targeting respiratory pathogens, pulmonary delivery of sa-mRNA lipid nanoparticles (LNPs) is particularly advantageous, enabling direct delivery to the infection site and induction of mucosal immunity. **Objective:** In this study, we evaluated the stability of sa-mRNA–LNPs under refrigerated and frozen conditions and developed a dry powder formulation suitable for inhalation, produced by freeze-drying followed by cryomilling with leucine. **Methods:** sa-mRNA–LNPs formulated in HEPES buffer with 20% (*w*/*v*) sucrose were stored for up to 8 weeks as liquid or freeze-dried samples at various temperatures (−80 °C, −20 °C, 4 °C, and 20 °C). Biological stability was assessed by transfection efficiency in HeLa cells, while physical stability was characterized by encapsulation efficiency, zeta potential, particle size, and polydispersity index. **Results:** Liquid formulations remained stable for at least 8 weeks at −80 °C and −20 °C but rapidly lost stability at 4 °C and 20 °C. Freeze-drying effectively preserved sa-mRNA–LNP functionality and structural integrity for up to 8 weeks at 4 °C, with only minor structural changes. Subsequent cryomilling in the presence of 4 wt-% leucine produced a respirable dry powder while retaining approximately 60% of the original sa-mRNA–LNP functionality. Although cryomilling induced some structural alterations, the remaining functional fraction remained stable during storage. The resulting powders displayed favorable aerosol performance for deep lung delivery, as demonstrated by cascade impaction (MMAD = 4.13 ± 0.26 µm). **Conclusions:** In conclusion, freeze-drying effectively preserved sa-mRNA–LNP integrity at 4 °C, whereas cryomilling with leucine produced a respirable dry powder suitable for pulmonary delivery, providing a foundation for globally accessible, needle-free sa-mRNA vaccines against respiratory diseases.

## 1. Introduction

The global health crisis caused by the COVID-19 pandemic has highlighted the urgent need for flexible, scalable, and rapidly deployable vaccine platforms. mRNA vaccines encapsulated in lipid nanoparticles (LNPs) have proven highly effective in this regard [1,2]. A major advantage of mRNA vaccines lies in their versatility: they can be synthesized quickly, adapted with relative ease, and manufactured at a scale compared to traditional vaccine platforms. Furthermore, mRNA vaccines elicit both humoral and cellular immune responses, in contrast to protein and inactivated vaccines, which primarily induce humoral immune responses. Encapsulation of the mRNA in LNPs further enhances their performance by improving cellular uptake, protecting mRNA from degradation, and facilitating endosomal escape, all of which are essential for robust protein expression [3]. Beyond COVID-19, mRNA–LNPs hold promise for a wide range of infectious diseases and therapeutic indications [4]. However, despite these successes, key challenges persist, particularly concerning the physicochemical stability of mRNA–LNPs, the limitations of intramuscular administration for mucosal pathogens, and the logistical constraints of cold-chain-dependent global distribution [1,3,5].

The success of conventional mRNA vaccines has paved the way for the clinical development of self-amplifying mRNA (sa-mRNA) vaccines as a next-generation platform [6]. sa-mRNA is an advanced form of mRNA technology that incorporates a viral replicase, enabling intracellular amplification of the antigen transcript. Upon delivery, the replicase synthesizes the complementary RNA strands that serve as templates for producing multiple copies of mRNA encoding the antigen. This process markedly increases and prolongs protein production, thereby prolonging antigen expression and thus enabling the induction of comparable immunogenicity at substantially lower doses than conventional mRNA. In vivo studies have indeed confirmed that sa-mRNA elicits potent immune responses [7,8,9,10,11]. Similarly to conventional mRNA, delivery of sa-mRNA is achieved through LNPs, typically composed of a combination of ionizable lipids, cholesterol, helper phospholipids, and PEG–lipids. Ionizable lipids mediate RNA encapsulation and endosomal escape, cholesterol and helper lipids provide structural support, and PEG–lipids control particle size and stability [3,12,13]. Subtle variations in lipid composition can significantly affect the transfection efficiency of both conventional mRNA and sa-mRNA [12,14,15]. Despite these design optimizations, sa-mRNA–LNPs remain highly sensitive to the degradation of both the sa-mRNA and the LNPs. sa-mRNA is highly susceptible to chemical degradation (e.g., hydrolysis or oxidation), while the lipid components undergo both chemical (e.g., hydrolysis or oxidation) and physical (e.g., aggregation or fusion) degradation [1,3]. This makes sa-mRNA–LNPs in aqueous solutions dependent on ultra-cold storage, posing major barriers to broad global distribution.

One promising strategy to address the stability constraints of conventional mRNA–LNP vaccines is freeze-drying [16]. Although the freeze-drying process imposes stress on both the mRNA and the LNPs during freezing and dehydration, the inclusion of sugars such as sucrose mitigates this damage by forming an (amorphous) glassy matrix. This matrix stabilizes the nanoparticles by replacing water molecules and immobilizing molecular structures, thereby protecting them from chemical and physical degradation [16]. The incorporation of lyoprotectants, such as sucrose or trehalose, has been shown to preserve both mRNA integrity and LNP structure during freeze-drying [15,16,17,18,19,20,21,22]. Recent studies have demonstrated that sucrose-based freeze-drying can effectively stabilize sa-mRNA–LNPs, achieving comparable stability to conventional mRNA–LNPs. Freeze-dried sa-mRNA–LNPs have been reported to retain both their structural integrity and biological activity during refrigerated storage for several months [23,24,25,26]. Notably, reconstituted sa-mRNA–LNP formulations maintained their immunogenic potency, eliciting strong and durable immune responses against target antigens [26]. However, not all sucrose-based freeze-dried formulations exhibit the same level of stability as freshly prepared sa-mRNA–LNPs [24], and a decrease in biological activity has been observed when stored at ambient temperatures [25].

While stability is a critical barrier to sa-mRNA vaccine deployment, the delivery route remains an equally important challenge. Current conventional mRNA–LNP vaccines are delivered via the inconvenient intramuscular route, inducing systemic immune responses. However, the mucosal immune response is limited upon intramuscular injection [27]. This is a critical drawback for respiratory pathogens, such as SARS-CoV-2, influenza, and respiratory syncytial virus, which primarily infect via mucosal surfaces of the respiratory tract. Mucosal immunity, particularly, secretory IgA and tissue-resident T cells, plays an important role in blocking infection at the site of viral entry and aerogene transmission [27]. Pulmonary vaccination, delivered intranasally or intratracheally, has demonstrated superior induction of local immune responses in preclinical studies [27]. Pulmonary delivery may therefore provide not only stronger protection against infectious disease but also reduced reinfection and onward transmission [28,29]. Although some studies developed conventional mRNA–LNPs for nebulization, this method faces challenges such as mRNA degradation, long administration times, and imprecise dosing [30,31,32,33]. In contrast, dry powder formulations offer a more stable and efficient alternative, offering a promising approach for pulmonary delivery of sa-mRNA–LNP vaccines or therapeutics [34].

To achieve sufficient deep lung deposition, drug formulations for inhalation must consist of dry powder particles with an aerodynamic diameter between 1 and 5 µm in order to produce an aerosol within that particle size range [35]. Spray drying and jet milling are commonly used methods to produce particles within this size range [36]. In spray drying, a liquid feed is atomized into a heated chamber, where rapid solvent evaporation produces fine solid particles [36]. However, low glass transition temperature (Tg) sugars (e.g., sucrose) pose challenges, as they become sticky under elevated relative humidity (RH) [37,38]. Although climate-controlled spray drying can mitigate this issue, it is costly and often yields powders with high electrostatic charge, complicating handling. Friis et al. demonstrated that trehalose-based spray drying of conventional mRNA–LNPs can produce stable dry powders, but this required a strictly controlled production environment (RH 6%) [39]. At higher humidities, spray-dried dry powder particles of low-Tg sugars exhibit stickiness, particle fusion, and crystallization. Jet milling, another particle-size-reduction method, relies on high-velocity particle collisions driven by compressed gas [36]. While effective for many materials, it is unsuitable for low-Tg sugars, as frictional heat can exceed the Tg, resulting in similar issues of stickiness, fusion, and crystallization [40]. Consequently, there remains a need for a particle engineering strategy that enables the production of inhalable dry powders without surpassing the Tg of the incorporated excipients.

To overcome this limitation, cryomilling of freeze-dried sucrose cakes can be utilized to maintain processing temperatures below the Tg, thereby preserving both the structural integrity of the LNPs and the biological activity of the sa-mRNA. Additionally, co-milling with excipients such as leucine can improve powder dispersibility and aerosol performance, ensuring consistent deposition in the deep lung [41]. Leucine is proposed to enhance powder dispersibility and aerosolization efficiency through the formation of a hydrophobic surface layer on dry powder particles. This surface modification reduces interparticle cohesive forces, thereby improving dispersibility and overall aerosol performance [42,43].

The resulting cryomilled dry powders require administration via an appropriate inhalation device, such as the Cyclops dry powder inhaler (DPI), which is specifically engineered for efficient powder dispersion, leading to effective deep lung delivery and offering scalability and disposable use [44,45]. Compared to nebulizers and metered-dose inhalers (MDIs), DPIs provide enhanced stability, portability, dose uniformity, and a lower carbon footprint [46,47]. Moreover, their needle-free, non-invasive design contributes to improved patient comfort and compliance [45,47].

To the best of our knowledge, in this study, we are the first to investigate both the stability of sa-mRNA–LNPs under refrigerated conditions and the production of an inhalable dry powder formulation using cryomilling. The first objective was to enhance the long-term stability of sa-mRNA–LNPs at 4 °C, thereby reducing reliance on ultra-cold storage. To achieve this aim, sa-mRNA–LNPs were freeze-dried with sucrose, which acts as a lyoprotectant by forming a glassy matrix that stabilizes the nanoparticles during freezing and dehydration, while also improving long-term stability at elevated storage temperatures. Building on this, the freeze-dried cakes were cryomilled to generate respirable particles suitable for pulmonary administration. To further optimize aerosol performance, leucine was incorporated as a dispersibility enhancer. Finally, the aerosolization behavior of the cryomilled powders was assessed using the Cyclops DPI to evaluate their suitability for deep lung deposition. By combining stabilization through freeze-drying with dry powder production via cryomilling, this study provides a novel strategy for formulating sa-mRNA–LNPs as stable, inhalable vaccines or therapeutics, thereby supporting their broader clinical translation and global accessibility.

## 2. Materials and Methods

### 2.1. Materials

Sucrose (S0389-500G) and HEPES molecular biology grade (H4034-100G) were obtained from Sigma-Aldrich (St. Louis, MO, USA). L-leucine (1711027.1653) was purchased from Ofipharma (Ter Apel, The Netherlands). HeLa cells were purchased from American Type Culture Collection (Manassas, VA, USA). Modified Eagle’s Medium (MEM) (31095-029), trypsin/EDTA, phosphate-buffered saline (PBS) (20012-019), trypan blue stain (0.4%) (15250-061), PrestoBlue (A13262), Quant-IT RiboGreen RNA assay kit (R11490), and fetal bovine serum (FBS) were obtained from Thermo Fisher Scientific (Waltham, MA, USA). D-luciferin was purchased from Perkin Elmer (Waltham, MA, USA).

### 2.2. Cells

HeLa cells were grown in MEM supplemented with 10% FBS. The cells were incubated at 37 °C and 5% CO_2_. The cells were harvested by using 1× 0.5% trypsin/EDTA. The cells were sub-cultured in a 1:5 ratio twice a week. Passages 10–20 were used for the experiments.

### 2.3. sa-mRNA Synthesis

The self-amplifying RNA was produced by in vitro transcription of a linear DNA template that was generated by PCR from an IVT PV01 plasmid using primers (forward: CAGGGTAATTAATACGACTCACTATAATG; reverse: TTTTTTTTTTTTTTTTTTTTTTTTTTTTTTTTTTTTTTTTGAAATATTAAAAACAAAATCCGATTC). The PV01 plasmid contains the Venezuelan Equine Encephalitis Virus strain TC-83 5′ UTR, 3′ UTR, non-structural proteins genes, and the luciferase gene. After the PCR, the DNA fragment was purified using silica spin columns (PCR & DNA Cleanup Kit, T1030S, New England Biolabs, Ipswich, MA, USA). The purified linear PCR DNA template was used for in vitro transcription (IVT) (AM1334, Thermo Fisher Scientific, Waltham, MA, USA) with co-transcriptional capping using CleanCap AU (CleanCap AU N711410, TriLink Biotechnologies, San Diego, CA, USA), according to the manufacturer’s protocol. The resulting mRNA was purified using an RNA Cleanup Kit (T2050L, New England Biolabs, Ipswich, MA, USA) and stored at −80 °C until further use.

### 2.4. Lipid Nanoparticle Formulation of the sa-mRNA

sa-mRNA-LNPs were formulated at an N/P ratio of 10 by rapidly mixing three volumes of sa-mRNA in sodium acetate buffer (pH 4.5) with one volume of an ethanolic lipid solution (100% ethanol) containing ALC-0315 (HY-138170, MedChemExpress, Monmouth Junction, NJ, USA), DMG-PEG2K (880151P, Avanti Polar Lipids, Alabaster, AL, USA), cholesterol (700100P, Avanti Polar Lipids, Alabaster, AL, USA), and DOPE (850725P, Avanti Polar Lipids, Alabaster, AL, USA) at a molar ratio of 50:1.5:38.5:10, as previously described in Cui et al.’s 2024 study [8]. Following formulation, the sa-mRNA–LNP suspension was directly dialyzed overnight against 20 mM HEPES buffer (pH 7.4; 15630080, Thermo Fisher Scientific, Waltham, MA, USA) using a dialysis cassette (66003, Thermo Fisher Scientific, Waltham, MA, USA) to remove ethanol and exchange the buffer. The yield is estimated to be approximately 60–80% of the input sa-mRNA. The resulting sa-mRNA–LNPs were transferred into polystyrene cuvettes and characterized by dynamic light scattering (Zetasizer Nano ZS, Malvern Panalytical, Burssels, Belgium) to determine the hydrodynamic diameter (reported as Z-average), polydispersity index (PDI), and zeta potential. sa-mRNA encapsulation efficiency was quantified using the Quanti-iT™ RiboGreen RNA assay kit (R11490, Thermo Fisher Scientific, Waltham, MA, USA) according to the manufacturer’s instructions and as described below. At Ghent University, the fluorescence was measured on a Cytation 5 plate reader (Agilent, Santa Clara, CA, USA) at 485 nm excitation and 525 nm emission in the absence and presence of 2% Triton X-100 (M236-10ML-5PK, VWR, Radnor, PA, USA), which disrupts the sa-mRNA–LNPs and releases encapsulated sa-mRNA.

### 2.5. Freeze–Thawing sa-mRNA–LNPs

The sa-mRNA–LNPs were transported from Ghent University to the University of Groningen within one day at 4 °C. The sa-mRNA–LNPs were diluted in 20% (*w*/*v*) sucrose in 20 mM HEPES buffer upon arrival. Sucrose at a concentration of 20% (*w*/*v*) was selected as the cryo- and lyoprotectant, as this level is used in the BNT162b2 COVID-19 vaccine and has been reported in previous studies to effectively stabilize mRNA–LNP formulations [16,24]. For the freeze–thaw experiments, the sa-mRNA–LNPs were put in RNase-free and sterile Eppendorf tubes. Each Eppendorf tube contained 3 µg sa-mRNA in 150 µL 20% (*w*/*v*) sucrose in 20 mM HEPES buffer (pH 7.4). The samples were stored at −80 °C, −20 °C, 4 °C, and 20 °C for 1 week, and at −80 °C and −20 °C for 8 weeks. The samples stored at −80 °C were used as the control samples. For the follow-up experiments, the sample was diluted in sterile 20 mM HEPES buffer (pH 7.4).

### 2.6. Freeze-Drying sa-mRNA–LNPs

The sa-mRNA–LNPs were produced at Ghent University and transported to the University of Groningen within one day at 4 °C. For the freeze-drying experiments, RNase-free and sterile glass vials with a loosely fitted lid to allow air exchange were used. For the stability study, 5 µg sa-mRNA was dissolved in 250 µL 20% (*w*/*v*) sucrose in 20 mM HEPES buffer (pH 7.4). For the cryomill experiments, 10 µg sa-mRNA was dissolved in 500 µL 20% (*w*/*v*) sucrose in 20 mM HEPES buffer (pH 7.4). The samples were put in the freeze-dryer (Salm and Kipp, Breukelen, the Netherlands) with a shelf temperature of −70 °C. After freezing, samples were freeze-dried at a pressure of 0.220 mbar and a shelf temperature of −35 °C for 33 h in the primary drying phase. Secondary drying was executed at 0.055 mbar and 20 °C for 21 h. The samples were stored at −80 °C, −20 °C, and 4 °C for 8 weeks. sa-mRNA–LNPs in 20% (*w*/*v*) sucrose stored at −80 °C as liquid formulations were used as the control samples.

### 2.7. Cryomilling of Freeze-Dried sa-mRNA–LNPs

Freeze-dried sucrose with sa-mRNA–LNPs was cryomilled using a cryomill (Retsch, Haan, Germany). For each run, 200 mg of freeze-dried sucrose containing 500 ng of sa-mRNA was transferred into a 5 mL cryomilling chamber composed of hardened steel and containing two 5 mm stainless-steel balls. This was performed in a nitrogen-filled glovebox maintaining 0% RH. Prior to milling, the freeze-dried cake was manually broken into smaller fragments using a spatula. Subsequently, 8.3 mg of leucine, equivalent to 4 wt-% of the total powder mass, was added to the chamber and manually blended into the powder using a spatula. After filling the chamber with freeze-dried sucrose and leucine, it was closed inside the glovebox to ensure that 0% RH was maintained within the cryomill chamber. Subsequently, the cryomill chamber was attached to the cryomill. The cryomill system was continuously cooled using an external liquid nitrogen tank to maintain cryogenic temperatures, thereby ensuring that the Tg of sucrose was not exceeded. The milling protocol consisted of an initial pre-cooling phase at 5 Hz (i.e., 5 oscillations of the grinding chamber per second) for 8 min, followed by a grinding phase at 30 Hz (i.e., 30 oscillations of the grinding chamber per second) for 2 min. After cryomilling, the system was allowed to heat up to ambient temperature before the chamber was removed. All handling of the cryomilled powder was performed inside a glovebox maintained under a nitrogen atmosphere at 0% RH to prevent moisture uptake. The RH in the glovebox was monitored using a thermohygrometer. The resulting cryomilled powder was transferred into an Eppendorf tube and vacuum sealed. Cryomilled sa-mRNA–LNPs were used for analysis directly upon milling, or they were stored at –80 °C for 4 weeks. sa-mRNA–LNPs in 20% (*w*/*v*) sucrose stored at −80 °C as liquid formulations were used as the control samples. The handling of the samples was performed at a low RH (<30%).

### 2.8. Modulated Differential Scanning Calorimetry

Freeze-dried sucrose was characterized using modulated differential scanning calorimetry (mDSC) with the Q2000 modulated differential scanning calorimeter (TA instruments, New Castle, DE, USA). Approximately 5–10 mg of freeze-dried sucrose was put in a Tzero aluminum pan for analysis. The samples were preheated at 60 °C for 30 min to allow residual moisture to evaporate, cooled to 10 °C, and then heated to 150 °C. The heating rate was set at 2 °C per minute, with a modulation amplitude of 0.318 °C per minute. The Tg was determined by identifying the inflection point on the reversing heat flow versus temperature curve. All samples were analyzed in triplicate.

### 2.9. Dynamic Vapor Sorption

The water sorption isotherms of freeze-dried sucrose were measured using a DVS-1000 gravimetric sorption analyzer (Surface Measurement Systems Limited, London, UK). Samples, with an initial mass of approximately 10 mg, were analyzed at 25 °C and ambient pressure. Water uptake by the sucrose blends was recorded across an RH range of 0% to 90%, in increments of 10%. The RH was increased once equilibrium was reached, defined as a change in mass of less than 0.5 μg over a 10 min period.

### 2.10. Dynamic Light Scattering

The particle size, polydispersity index (PDI), and zeta potential of the sa-mRNA–LNPs were determined at the University of Groningen by dynamic light scattering (DLS) using a Mobius and Atlas instrument (Wyatt Technology, Santa Barbara, CA, USA). For freeze–thaw stability studies, 5 µL of sa-mRNA–LNP suspension was diluted in 495 µL of 20 mM HEPES buffer (pH 7.4). For freeze-dried samples, 10 mg of freeze-dried powder was reconstituted in 833 µL of 20 mM HEPES buffer (pH 7.4). Similarly, for the cryomilled powders, 10 mg of material was reconstituted in 833 µL of 20 mM HEPES buffer (pH 7.4). These volumes were selected to ensure that all measured samples contained equivalent concentrations of sa-mRNA–LNPs. Analyses were performed with 10 acquisitions per sample, each with an acquisition time of 10 s. Measurements were performed in triplicate.

### 2.11. Nanoparticle Tracking Analysis

For the cryomilled samples, the hydrodynamic particle size distribution and concentration of the sa-mRNA–LNPs were assessed using nanoparticle tracking analysis (NTA), which relies on light scattering (Nanosight NS300, Worcestershire, UK). For each sample, three video recordings of 60 s were captured using a flow cell. The videos were processed using the NTA software version 3.4.4. The camera level was set to 16, and the detection threshold was adjusted to 7. Frozen sa-mRNA–LNPs at −80 °C were diluted 5000× with MQ water before analysis. For the sa-mRNA–LNPs that were freeze-dried or freeze-dried and cryomilled, a total of 10 mg was put in an Eppendorf tube. A total of 500 µL MQ water was added to the Eppendorf tube to redisperse the sample. Consequently, this was 1000× diluted. From the acquired videos, the mode particle size, concentration, and the size distributions (span) were determined. The span was calculated using the formula below. Measurements were performed in triplicate.

$$span=\frac{\left(X90-X10\right)}{X50}$$

### 2.12. Encapsulation Efficiency

The encapsulation efficiency (EE) of the sa-mRNA–LNPs was determined using the Quant-iT™ RiboGreen™ RNA Assay Kit (R11490, Thermo Fisher Scientific, Waltham, MA, USA). All of the materials used were nuclease free. A 1× Tris-EDTA (TE) buffer was prepared. A 1× TE buffer containing 2% (*v*/*v*) Triton X-100 (Thermo Fisher Scientific, Waltham, MA, USA) was prepared by mixing 98 mL of 1× TE buffer with 2 mL of Triton X-100. The RiboGreen RNA standard was diluted 200-fold in 1× TE buffer to yield a working RNA solution of 2 µg/mL. The RNA concentration was verified spectrophotometrically using a NanoDrop (Thermo Fisher Scientific, Waltham, MA, USA) at an absorbance of 260 nm (A260). A calibration curve ranging from 20 to 1000 ng/mL RNA was prepared in duplicate on the 96-well plate. For sample analysis, 233 µL of 1× TE buffer and 16.7 µL of sa-mRNA–LNP dispersion (corresponding to 2 µg/mL sa-mRNA) were added to the first set of wells. To assess total and non-encapsulated sa-mRNA, 50 µL of sa-mRNA–LNP dispersion was diluted with either 50 µL 1× TE buffer (for intact LNPs) or 50 µL 1× TE buffer containing 2% Triton X-100 (for disrupted LNPs). After gentle mixing, the plate was incubated for 10 min at 37 °C, followed by 5 min at ambient temperature to ensure complete disruption and RNA release in the Triton-containing samples. Subsequently, 100 µL of the RiboGreen working solution was added to each well and incubated for 2 min at room temperature, protected from light. Fluorescence was measured using a Synergy HT plate reader (BioTek Instruments Inc., Winooski, VT, USA) with excitation and emission wavelengths of 480 and 520 nm, respectively. Encapsulation efficiency (EE%) was calculated using the following equation:

$$\frac{F\left(triton\;treated\right)-F(untreated)}{F(triton\;treated)}\times 100\%$$

with F (triton treated) as the fluorescence intensity of LNPs lysed with triton (representing total RNA content) and F (untreated) as the fluorescence intensity of intact sa-mRNA–LNPs without triton treatment (representing unencapsulated RNA). Both signals are reduced by the background signal. Results are expressed as a percentage relative to the EE of freshly prepared sa-mRNA–LNPs. All measurements were performed in triplicate.

### 2.13. In Vitro Transfection Efficiency with HELA Cells

HeLa cells were cultured in a 24-well plate with 0.05 × 10^6^ cells/well for 24 h. After 24 h, the cells were washed twice with PBS. Subsequently, the cells were transfected with 500 ng sa-mRNA–LNPs/well in MEM culture medium for 24 h. After 24 h, cells were washed with PBS, underwent trypsinization with 200 µL trypsin/EDTA for 5 min, and, lastly, 300 µL MEM with 10% FBS was added. In order to measure the transfection efficiency, 180 µL single-cell suspension was transferred to a white 96-well plate and 20 µL 1× D-luciferin was added. The plate was covered with aluminum foil and after 2 h of incubation at 37 °C, the bioluminescence (578 nm) was measured with the plate reader (Synergy HT, BioTEK, Winooski, VT, USA). In order to measure the viability of the cells, the PrestoBlue Assay for Cell Viability was used. For this assay, 90 µL single-cell suspension was transferred to a black 96-well plate. Consequently, 10 µL PrestoBlue (A13261, Thermo Fisher Scientific, Waltham, MA, USA) was added to each well in a dark room. The plate was covered with aluminum foil to ensure darkness, and the plate was incubated for 2 h at 37 °C. The fluorescence was measured at a wavelength of 530/590 nm (excitation/emission) with the plate reader (Synergy HT, BioTEK, Winooski, VT, USA). Measurements were performed in triplicate.

### 2.14. Scanning Electron Microscopy

The morphology of the cryomilled powder particles was examined using scanning electron microscopy (SEM) with a JSM 6460 instrument (Jeol, Tokyo, Japan). For visualization, the powder was affixed to aluminum specimen mounts using carbon-based double-sided adhesive tape. This was performed in a glove box set at 0% RH. Prior to imaging, a conductive gold–palladium layer, approximately 10 nm in thickness, was deposited using a JFC-1300 automatic fine coater (Jeol, Tokyo, Japan) to enhance surface conductivity. SEM analysis was conducted under vacuum at an accelerating voltage of 10 kV, with a working distance set to 10 mm and a spot size parameter of 25.

### 2.15. Primary Particle Size Analysis

The geometric particle size distribution of cryomilled freeze-dried sa-mRNA–LNPs formulated with sucrose and leucine was measured by laser diffraction. A RODOS dry powder disperser, operating at a pressure of 3 bar, was coupled to a HELOS BF laser diffraction unit (Sympatec, Clausthal-Zellerfeld, Germany) equipped with an R3 lens (100 mm focal length). Roughly 10 mg of sample was loaded into the dispersion funnel. Data collection began when the optical density reached 0.2% on channel 30, with each analysis running for 3 s. Particle size distribution values (X50) were calculated using the Fraunhofer approximation to describe the size profile of the particles. All measurements were performed at an RH below 30%.

### 2.16. Dispersion Efficiency of Cryomilled sa-mRNA–LNPs Using the CYCLOPS DPI

The aerosolization performance of cryomilled sa-mRNA–LNPs formulated with sucrose was investigated using the Cyclops dry powder inhaler (PureIMS, Roden, The Netherlands), with a 4.75 mm discharge hole. Particle size distribution under simulated inhalation conditions was assessed with a HELOS BF laser diffraction system fitted with the Inhaler2000 attachment (Sympatec, Clausthal-Zellerfeld, Germany). For each trial, 25 mg of the formulation was placed into the Cyclops dosing compartment. A reference measurement was completed before each test sequence. During analysis, airflow conditions were maintained at a pressure drop of 4 kPa over a 5 s interval. Data recording began when the optical signal reached 0.2% intensity on channel 30. After testing, the inhaler device was reweighed to estimate residual powder. From the diffraction data, the particle size (X50) was calculated. Additionally, the fine particle fraction (FPF), defined as the percentage of material with an aerodynamic diameter ≤ 5 µm, was used to evaluate suitability for lung deposition. All measurements were performed at an RH below 30%. All measurements were conducted in triplicate.

### 2.17. Cascade Impaction Analysis

The aerodynamic particle size distribution of cryomilled sa-mRNA–LNPs incorporated in sucrose was determined using cascade impaction analysis. This assessment was conducted with the Next Generation Impactor (NGI, MSP Corp. Shoreview, MN, USA), following the guidelines outlined in the European Pharmacopoeia for Apparatus E (Chapter 2.9.18). The NGI setup includes a standard USP/Ph. Eur. induction port simulating the oropharyngeal region, seven collection stages for aerodynamic fractionation, and a micro-orifice collector (MOC) to capture submicron particles. For each test, 25 mg of cryomilled sa-mRNA–LNP powder in sucrose was loaded into the dosing cup of the Cyclops dry powder inhaler (PureIMS, Roden, The Netherlands) in a glove box at 0% RH. The NGI was placed in an atmosphere with an RH below 30%. Whatman glass fiber filters (pore size: 0.2 µm; diameter: 50 mm) wetted with 1 mL of distilled water were placed on stages 1 through 7 to minimize particle bounce. Residual ultra-fine particles were collected using a wash bottle placed downstream of the NGI. The Cyclops inhaler was connected to the NGI via a sealed mouthpiece, and a pressure drop of 4 kPa was applied for 5.5 s, resulting in an airflow rate of 50 L/minute. Post-dispersion, the inhaler and the induction port were rinsed three times with distilled water, and the solvent was collected in a collection vial. The impactor stages and MOC were rinsed three times as well. Distilled water was put in the impactor stages and MOC, and after 30 min, the solvent from the cups was transferred into a collection vial. Fresh distilled water was then added to the cups, and after another 15 min, the solution was transferred again to the same vial. This process was repeated once more, resulting in a total soaking time of 60 min to ensure complete dissolution and maximal recovery of the formulation. The eluates were collected into pre-weighed containers for gravimetric analysis. The procedure was carried out in triplicate.

### 2.18. Sample Analysis Cascade Impaction Analysis

The dry powder collected from each stage of the NGI was quantified using the colorimetric Anthrone assay, with which the concentration of carbohydrates in aqueous solutions can be quantified [48]. A 2 mg/mL Anthrone reagent (A19118.14, Sigma-Aldrich, St. Louis, MO, USA) was prepared in concentrated analytical-grade sulfuric acid (76051908.1000, Boom BV, Meppel, The Netherlands). Calibration standards ranging from 0 to 200 µg/mL were prepared using the same cryomilled sa-mRNA–LNP sucrose formulation employed in the cascade impaction study. For quantification, 0.5 mL of each sample or standard was mixed with 2 mL of the Anthrone solution. The mixtures were vortexed and subsequently heated in a boiling water bath for 10 min. Glass beads were placed on top of each reaction tube to minimize solvent loss. After heating, the tubes were allowed to cool to ambient temperature. Then, 1 mL of the reaction mixture was transferred to a disposable cuvette, and absorbance was measured at a wavelength of 620 nm using a UV–Vis spectrophotometer (Genesys, Thermo Fisher Scientific, Waltham, MA, USA). The amount of deposited powder was determined by interpolation from the calibration curve. Powder recovery within the NGI ranged from 90% to 100%. Powder deposition across NGI stages was used to calculate the mass median aerodynamic diameter (MMAD) and FPF for nominal, emitted, and delivered doses. These values were derived using non-linear regression analysis, as previously described [49]. The FPF was defined as the percentage of particles with an aerodynamic diameter of ≤5 µm.

### 2.19. Statistical Analysis

All results are reported as mean values with standard deviation (SD). Sample sizes and replicate numbers are specified in the related figure legends. Comparisons involving more than two groups were evaluated using one-way analysis of variance (ANOVA), followed by Dunnett’s post hoc procedure for multiple comparisons or Tukey’s multiple comparisons test. A *p*-value below 0.05 was interpreted as significant. For comparisons between two groups, an unpaired Student’s *t*-test was applied. All analyses were conducted using GraphPad Prism, version 10 (GraphPad Software).

## 3. Results and Discussion

### 3.1. Production and Characterization of the sa-mRNA–LNPs

Directly after formulation of the sa-mRNA–LNPs, their size, zeta potential, and EE were determined at Ghent University. The resulting sa-mRNA–LNPs dispersed in 20 mM HEPES buffer (pH 7.4) had a mean diameter of 131.22 ± 11.60 nm, a negative zeta potential of −4.97 ± 6.24 mV, and an average EE of 96.79 ± 1.94%. After the arrival of the sa-mRNA-LNPs at the University of Groningen, these parameters were rechecked and gave similar results.

### 3.2. Frozen sa-mRNA–LNPs Are Stable at −20 °C for up to 8 Weeks

sa-mRNA–LNPs generally require storage at −80 °C to preserve long-term stability. While effective, such ultra-low-temperature conditions are financially and logistically demanding. To explore more practical storage strategies, we examined whether the addition of sucrose would enable sa-mRNA–LNPs to retain stability at higher temperatures (−20 °C, 4 °C, and 20 °C) for up to 8 weeks. The 8-week time point was selected because it represents a distribution-relevant storage duration, particularly for deployment to remote regions where access to ultra-low-temperature storage facilities is limited. Sa-mRNA–LNPs formulated with 20% (*w*/*v*) sucrose and stored at −80 °C exhibited transfection efficiencies comparable to freshly prepared particles. Therefore, these −80 °C samples were used as the reference condition. Transfection efficiencies measured after storage at −20 °C, 4 °C, and 20 °C were normalized to this reference to enable direct comparison of stability across temperatures.

**Figure 1:**
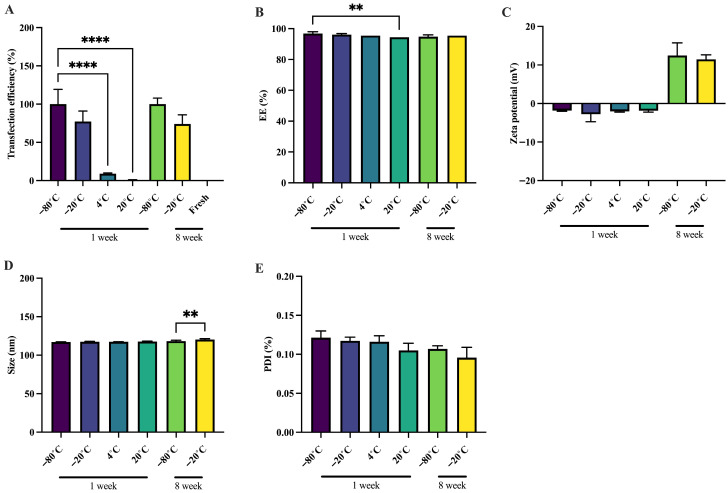
sa-mRNA–LNPs in liquid formulations are biologically and physically stable for up to 8 weeks when stored at −20 °C with 20% (*w*/*v*) sucrose. sa-mRNA–LNPs were stored at −80 °C, −20 °C, 4 °C, and 20 °C in 20% (*w*/*v*) sucrose and 20 mM HEPES buffer. (**A**) Transfection efficiency in HeLa cells as a percentage (%) of the sa-mRNA–LNPs frozen at −80 °C with 20% (*w*/*v*) sucrose. (**B**) Encapsulation efficiency (EE) as a percentage (%) relative to the EE of freshly prepared sa-mRNA–LNPs. The SD of 4 °C and 20 °C is very small and therefore not visible in the graph. (**C**) Zeta potential (mV). (**D**) Size (nm). (**E**) Polydispersity index (PDI). *N* = 3. One-way Anova. ** *p* < 0.01 and **** *p* < 0.0001.

The biological stability of sa-mRNA–LNPs was assessed by measuring the transfection efficiency using HeLa cells. sa-mRNA–LNPs stored at −20 °C in the presence of sucrose maintained their transfection efficiency for up to 8 weeks. By contrast, samples stored at 4 °C and 20 °C exhibited a significant decline in transfection efficiency within 1 week, indicating rapid degradation of the sa-mRNA (Figure 1A). Due to this rapid loss of activity, storage at 4 °C and 20 °C was not pursued in the extended 8-week study. Cell viability was unaffected across all conditions and time points (Figure S1A).

The physical stability of sa-mRNA–LNPs was assessed by measuring EE, zeta potential, particle size, and PDI. The EE of the sa-mRNA–LNP formulations remained unaffected during storage at −20 °C for up to 8 weeks. At 4 °C, the EE was maintained for 1 week without significant loss. In contrast, a slight but significant decrease in the EE was observed after 1 week of storage at 20 °C. Nevertheless, the EE remained above 94% under all tested conditions, indicating that the majority of the sa-mRNA remained encapsulated within the LNPs, even at elevated storage temperatures (Figure 1B). The zeta potential of the LNP formulations remained constant across all storage conditions for up to 1 week, indicating preserved physical stability of the nanoparticles. After 8 weeks of storage, a pronounced increase in zeta potential was observed compared to 1 week (Figure 1C). This change is likely associated with structural rearrangements occurring during the freezing or thawing of the sa-mRNA–LNPs, rather than during storage itself. This finding is consistent with previous reports linking elevated zeta potential values to surface rearrangements in lipid nanoparticles [24]. The particle size of the LNPs remained stable for up to 1 week at all tested temperatures. However, after 8 weeks, samples stored at −20 °C exhibited a non-relevant but significant increase in size compared to those stored at −80 °C (Figure 1D). The PDI remained unchanged across all storage temperatures and time points, confirming consistent size distribution of the LNP formulations (Figure 1E).

These results showed that samples stored at 4 °C and 20 °C retained their physical integrity and exhibited a high EE, yet complete loss of functional of sa-mRNA was observed within 1 week, reflecting a loss of chemical integrity. These findings indicate that while the LNPs remain intact and encapsulate sa-mRNA at non-frozen temperatures, they do not prevent sa-mRNA degradation. The instability of sa-mRNA under these conditions is likely driven primarily by hydrolysis, in which residual water facilitates nucleophilic cleavage of the phosphodiester backbone [50,51]. Additional degradation pathways, including oxidation, nucleobase–lipid adduct formation, and transesterification, may also contribute to RNA breakdown [50,51,52]. In conclusion, sa-mRNA–LNPs formulated with 20% (*w*/*v*) sucrose demonstrated robust biological and physical stability when stored at −20 °C for at least 8 weeks.

### 3.3. Freeze-Dried sa-mRNA–LNPs Retain Most of Their Stability for up to 8 Weeks at 4 °C

Although sa-mRNA–LNPs remain stable at −20 °C, achieving stability at 4 °C would substantially simplify storage and distribution, thereby improving global accessibility of sa-mRNA–LNP vaccines or therapeutics^1^. To accomplish this, the sa-mRNA–LNPs were freeze-dried with 20% (*w*/*v*) sucrose to improve their stability at 4 °C. The freeze-dried sucrose sa-mRNA–LNP samples formed a porous, white cake, indicating a successful freeze-drying process. The freeze-dried sa-mRNA-LNPs were stored for 8 weeks at −80 °C, −20 °C, and 4 °C. Non-freeze-dried sa-mRNA–LNPs stored at −80 °C with 20% (*w*/*v*) sucrose served as the control, and the transfection efficiency values obtained after storage upon freeze-drying at −80 °C, −20 °C, and 4 °C were normalized to these control samples to facilitate comparison.

**Figure 2:**
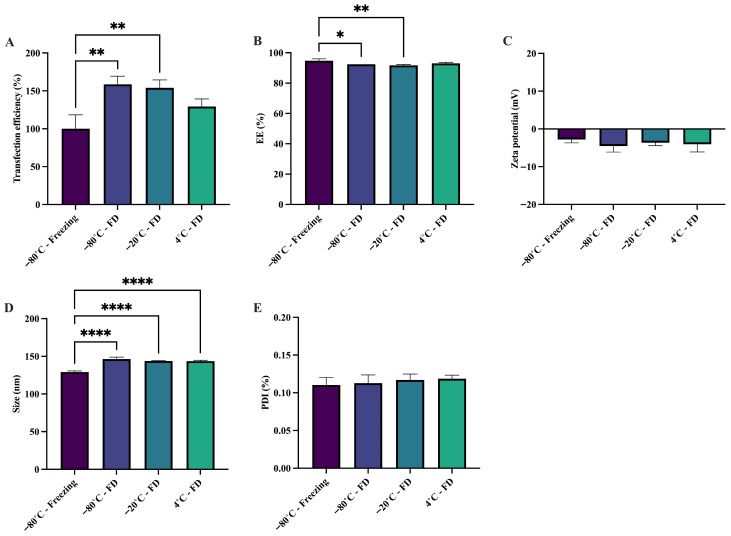
Freeze-dried sa-mRNA–LNPs are biologically and physically stable for up to 8 weeks at 4 °C with 20% (*w*/*v*) sucrose. sa-mRNA–LNPs were frozen and stored at −80 °C in 20% (*w*/*v*) sucrose and 20 mM HEPES buffer as a control. sa-mRNA–LNPs were freeze-dried with 20% (*w*/*v*) sucrose and 20 mM HEPES and stored at −80 °C, −20 °C, and 4 °C. (**A**) Transfection efficiency in HeLa cells as a percentage (%) of the sa-mRNA–LNPs frozen at −80 °C with 20% (*w*/*v*) sucrose. (**B**) Encapsulation efficiency (EE) as a percentage (%) relative to the EE of freshly prepared sa-mRNA–LNPs. (**C**) Zeta potential (mV). (**D**) Size (nm). (**E**) Polydispersity index (PDI). FD = freeze-dried. *N* = 3. One-way Anova. * *p* < 0.05, ** *p* < 0.01, and **** *p* < 0.0001.

To assess the biological stability of sa-mRNA following freeze-drying, the transfection efficiency was evaluated. Storage at −80 °C, −20 °C, and 4 °C upon freeze-drying preserved transfection efficiency for up to 8 weeks, although a significant increase in activity was observed in samples stored at −80 °C and −20 °C upon freeze-drying (Figure 2A). In contrast, cell viability decreased significantly across all storage conditions after freeze-drying (Figure S1). The increased transfection efficiency observed after freeze-drying suggests enhanced cytosolic availability of saRNA, potentially driven by sucrose-mediated promotion of vesicle trafficking, fusion, and endosomal processing, as reported previously [53,54]. While this enhanced intracellular delivery results in higher reporter expression, it may also increase the activation of innate RNA-sensing pathways due to elevated cytosolic RNA levels. At the relatively high in vitro transfection doses used, even modest improvements in uptake or endosomal escape may therefore contribute to the reduction in cell viability.

Physical stability of sa-mRNA–LNPs was examined by measuring the EE, zeta potential, particle size, and PDI. The EE remained stable after freeze-drying and during storage at 4 °C for 8 weeks. However, a small, but significant, reduction in EE was observed after 8 weeks of storage upon freeze-drying at both −80 °C and −20 °C. Despite this decline, EE values remained above 90%, indicating that the majority of sa-mRNA remained entrapped within the LNPs at all storage conditions (Figure 2B). These results suggest that the LNPs largely retain their encapsulation capacity when freeze-dried and stored up to 4 °C. The zeta potential and PDI were consistent across all storage conditions after freeze-drying for up to 8 weeks (Figure 2C,E). In contrast, particle size increased slightly but significantly following freeze-drying. However, sizes remained below 150 nm (Figure 2D), which is consistent with previous observations [23,25].

Although the freeze-drying process itself can impose stress on sa-mRNA–LNPs, the inclusion of lyoprotectants such as sucrose mitigates these effects and helps preserve the structural and functional integrity of sa-mRNA–LNPs [55]. In conclusion, freeze-drying of sa-mRNA–LNPs with 20% (*w*/*v*) sucrose allows storage at 4 °C for up to 8 weeks with minimal loss of physical and biological stability. This strategy may enhance the accessibility and practicality of sa-mRNA-based therapeutics by reducing dependence on ultra-low-temperature storage.

### 3.4. Cryomilled sa-mRNA–LNPs Retain Most of Their Stability After Storage for 4 Weeks

Freeze-drying with sucrose as a stabilizer proved effective in preserving the stability of sa-mRNA–LNPs at 4 °C for up to 8 weeks. Given the potential of sa-mRNA–LNPs as vaccines or therapeutics for respiratory diseases, pulmonary delivery represents an attractive route of administration. However, formulating these nanoparticles into inhalable dry powders poses specific challenges. Although spray drying and jet milling are widely applied methods for producing inhalable powders, their compatibility with sucrose is limited due to the excipient’s low Tg and hygroscopic nature. To better understand these limitations, the solid-state properties of freeze-dried sucrose were characterized using mDSC and DVS. mDSC analysis revealed a Tg of 73.13 ± 1.45 °C (Figure S2), while DVS confirmed the hygroscopicity of sucrose, with crystallization occurring at 50% RH at 25 °C (Figure S3). In light of these properties, cryomilling was explored as an alternative approach to generate inhalable dry powders. Milling under cryogenic conditions allows a reduction in the particle size of the freeze-dried cake while keeping the temperature far below the Tg of sucrose, which is critical for maintaining the stability of sa-mRNA–LNPs. This strategy offers a promising route for producing inhalable dry powders containing sa-mRNA–LNPs without compromising their physical stability. Using this strategy, freeze-dried cakes of sa-mRNA–LNPs with 20% (*w*/*v*) sucrose were cryomilled and stored at −80 °C for up to 4 weeks. Non-freeze-dried sa-mRNA–LNPs stored at −80 °C with 20% (*w*/*v*) sucrose were used as controls, and the transfection efficiency values obtained after freeze-drying, cryomilling, and subsequent storage were normalized to these controls to facilitate direct comparison.

**Figure 3:**
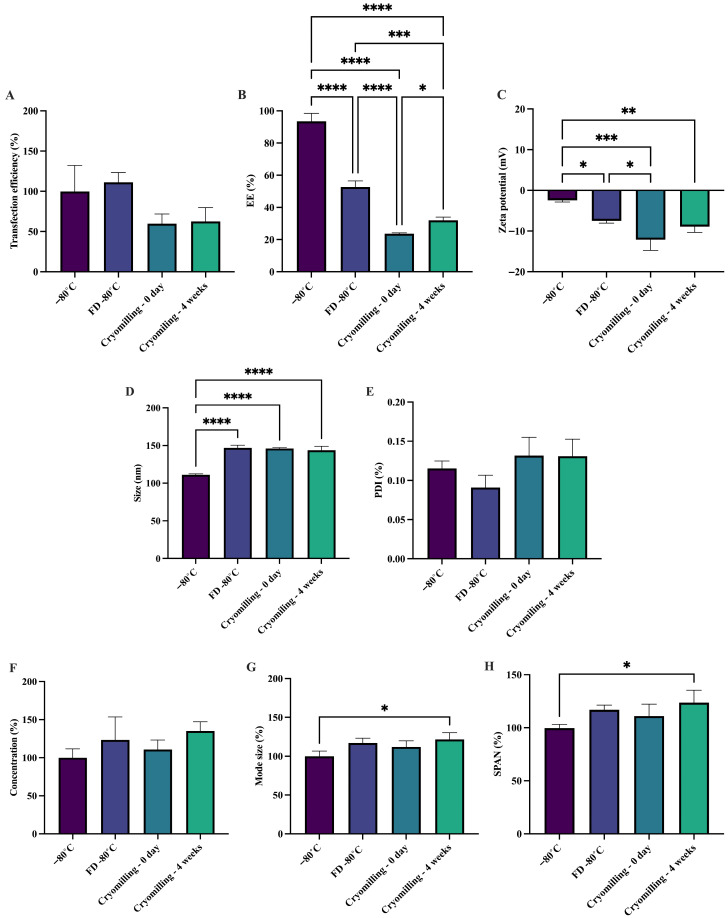
Cryomilled sa-mRNA–LNPs are functional for up to 4 weeks. sa-mRNA–LNPs were stored at −80 °C with 20% (*w*/*v*) sucrose and 20 mM HEPES buffer, freeze-dried in 20% (*w*/*v*) sucrose and 20 mM HEPES, and freeze-dried and cryomilled in 20% (*w*/*v*) sucrose and 20 mM HEPES buffer. Stability measurements were performed immediately upon cryomilling and after 4 weeks of storage at −80 °C. (**A**) Transfection efficiency (%) shown as a percentage of sa-mRNA–LNP stored at −80 °C. (**B**) Encapsulation efficiency (EE) shown as a percentage (%) relative to the EE of freshly prepared sa-mRNA–LNPs. (**C**) Zeta potential (mV). (**D**) Size (nm). (**E**) Polydispersity index (PDI). (**F**) Concentration (%) measured with NTA and shown as a percentage of sa-mRNA–LNPs stored at −80 °C. (**G**) Mode size (%) measured with NTA and shown as a percentage of sa-mRNA–LNPs stored at −80 °C. (**H**) SPAN (%) measured with NTA and shown as a percentage of sa-mRNA–LNPs stored at −80 °C. *N* = 3. One-way Anova. * *p* < 0.05, ** *p* < 0.01, *** *p* < 0.001, and **** *p* < 0.0001. *N* = 3.

Cryomilling exposes sa-mRNA–LNPs to multiple stresses, including shear stress, which almost inevitably may compromise their stability. Therefore, the primary objective was to determine whether the sa-mRNA–LNPs could withstand the cryomilling process. To evaluate both biological and physical stability, samples were analyzed immediately after cryomilling and following 4 weeks of storage at −80 °C. Cryomilling caused an immediate reduction of approximately 40% in transfection efficiency, indicating substantial loss of functional sa-mRNA during processing (Figure 3A). However, no further decline in transfection efficiency was observed after 4 weeks of storage, demonstrating that the processing step, rather than storage, was responsible for the initial loss of activity. Similarly, cell viability decreased significantly directly after cryomilling but remained unchanged during subsequent storage (Figure S1).

Physical stability of the sa-mRNA–LNPs was evaluated by measuring the EE, zeta potential, particle size, and PDI. The EE decreased significantly after freeze-drying compared to the control samples, with an additional reduction observed following cryomilling. Storage for 4 weeks post-cryomilling did not further decrease the EE, suggesting that the initial loss occurs during processing steps rather than storage (Figure 3B). The zeta potential decreased significantly after freeze-drying and cryomilling. However, values remained within −10 mV to +10 mV, a range typically regarded as approximately neutral [56] (Figure 3C). Particle size increased significantly in both freeze-dried and cryomilled samples compared to the frozen control, but no further increase was observed between freeze-dried and cryomilled formulations, indicating that cryomilling did not further alter particle size (Figure 3D). PDI values remained unchanged across all conditions (Figure 3E). Collectively, these findings suggest that while cryomilling compromises EE, the overall physicochemical properties of the sa-mRNA–LNPs remain largely preserved.

These results showed that sa-mRNA–LNPs withstand cryomilling to a large extent, maintaining 60% of their biological stability and maintaining their size and PDI. Nevertheless, changes in particle characteristics were observed upon cryomilling, most notably, a reduction in EE and zeta potential. A decrease in EE suggests partial leakage of sa-mRNA from the LNPs, which may arise from mechanical stresses induced during cryomilling. The decline in zeta potential further points to alterations in the surface composition of the LNP, which could influence particle–particle interactions and colloidal stability. These observations are consistent with previous reports on the formulation of conventional mRNA–LNPs into inhalable dosage forms, particularly through nebulization or spray drying. For instance, nebulization has been shown to increase the particle size, zeta potential, and PDI, and decrease the EE of conventional mRNA–LNPs [30,31,33]. Similarly, spray drying of conventional mRNA–LNPs resulted in reduced EE [39]. These recurring observations across various inhalable particle engineering techniques highlight the sensitivity of conventional mRNA–LNPs to mechanical and interfacial stresses. It is likely that sa-mRNA–LNPs exhibit similar behavior, as they share comparable structural and compositional characteristics with conventional mRNA–LNPs. Importantly, studies consistently demonstrated that despite physical changes, the mRNA remained functionally active and capable of protein expression [30,31,39]. This distinction emphasizes that physical alterations in LNP characteristics do not necessarily translate into chemical degradation of the RNA cargo.

To further investigate physical stability, NTA was performed. Particle concentrations were consistent across freeze-dried, cryomilled, and stored samples, indicating minimal particle loss or aggregation (Figure 3F and S4). The mode particle size and size distribution (span) were comparable immediately after freeze-drying and cryomilling, suggesting no major immediate changes in LNP morphology, although subtle structural alterations cannot be ruled out. However, after 4 weeks of storage post-cryomilling, both mode size and span increased significantly, pointing to gradual particle aggregation over time (Figure 3G,H and S4). In conclusion, sa-mRNA–LNPs retain approximately 60% of their transfection efficiency following cryomilling and 4 weeks of storage at −80 °C. The size and PDI remained unchanged throughout processing and storage. In contrast, EE and zeta potential were reduced immediately after cryomilling but remained stable during subsequent storage. Together, these findings indicate that although cryomilling affects a fraction of the sa-mRNA–LNPs, the remaining ~60% of intact particles successfully withstand the cryomilling process and maintain their stability during subsequent storage.

### 3.5. Cryomilled sa-mRNA–LNPs Are Suitable for Deep Lung Deposition

**Figure 4:**
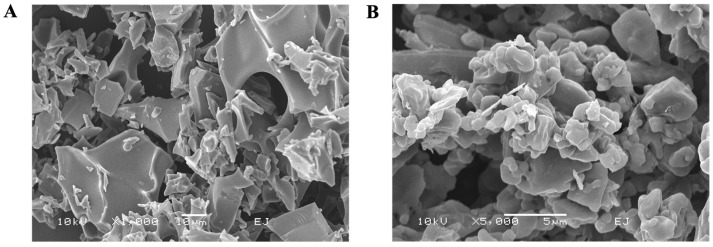
Cryomilled freeze-dried sa-mRNA–LNPs in sucrose appear as small, flake-like structures. (**A**) Freeze-dried sa-mRNA–LNPs in sucrose before milling. Magnification 1000×. (**B**) Cryomilled freeze-dried sa-mRNA–LNPs in sucrose after milling. Magnification 5000×.

**Figure 5:**
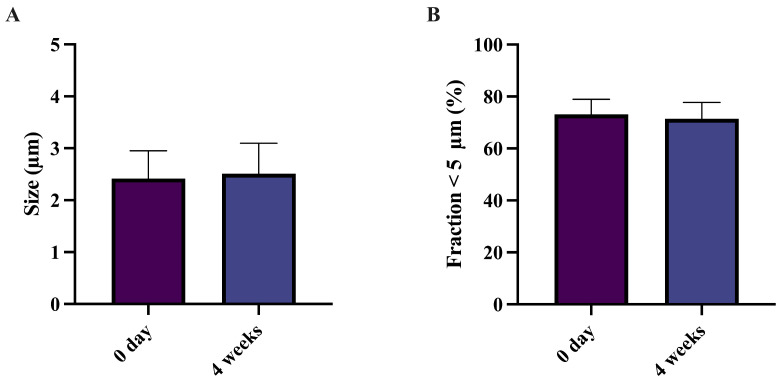
Cryomilled freeze-dried sa-mRNA–LNPs with sucrose and 4% leucine have a primary particle size suitable for deep lung deposition. (**A**) Size (µm) of cryomilled freeze-dried sa-mRNA–LNPs in sucrose directly after cryomilling and after 4 weeks of storage at −80 °C. (**B**) Fraction < 5 µm (%) of cryomilled freeze-dried sa-mRNA–LNPs in sucrose directly after cryomilling and after 4 weeks of storage at −80 °C. Student’s *t*-test. *N* = 3.

To facilitate the development of sa-mRNA–LNPs as an inhalable vaccine, it is essential to evaluate their deposition and distribution in the deep lung. The sa-mRNA–LNPs were first freeze-dried and subsequently cryomilled with 4 wt-% leucine. Leucine was used as an excipient, since it has been shown to have dispersion-improving capabilities [57]. Scanning electron microscopy images revealed that the cryomilled powders consisted of small, flake-like particles with reduced size compared to the intact freeze-dried cake (Figure 4A,B). Geometric primary particle size distribution, analyzed by RODOS, demonstrated an X50 of 2.42 ± 0.44 µm and fraction ≤ 5 µm of 73.1 ± 4.8% immediately after cryomilling (Figure 5A,B). After 4 weeks of storage, particle size remained stable, with an X50 of 2.51 ± 0.48 µm and a fraction ≤ 5 µm of 71.5 ± 5.1% (Figure 5A,B). Collectively, these results indicate that the primary particle size of cryomilled sa-mRNA–LNPs formulated with sucrose meets the criteria for effective deep lung deposition, supporting their potential for pulmonary vaccine delivery.

To enable effective delivery of sa-mRNA–LNPs to the lungs, a suitable inhalation device is required. The Cyclops DPI was identified as an appropriate device for administering dry powder formulations to the deep lungs. Laser diffraction analysis demonstrated an FPF of 69 ± 9% with an X50 of 3.04 ± 0.44 µm (Table 1). Additionally, only 17 ± 2% of the powder remained within the Cyclops DPI after simulated inhalation, indicating efficient powder dispersion and emission (Table 1). As a result, from the initial 25 mg loaded into the inhaler, 15.47 ± 2.28 mg of the emitted powder had a particle size below 5 µm, aligning with the requirements for deep lung deposition (Table 1). These findings confirm that cryomilled sa-mRNA–LNPs formulated with sucrose and leucine have appropriate particle size characteristics for pulmonary delivery when administered using a DPI.

**Figure 6:**
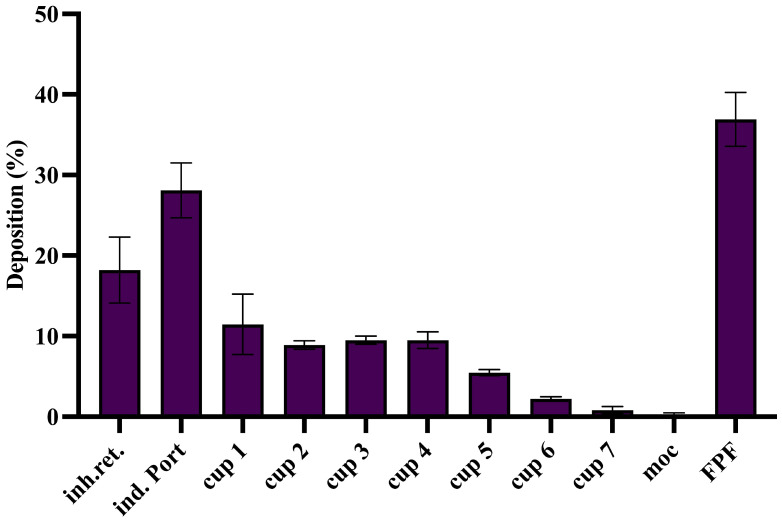
Cascade impaction analysis showed that cryomilled freeze-dried sa-mRNA–LNPs with sucrose and 4% leucine are suitable for deep lung deposition. FPF = fine particle fraction of the emitted dose. Inh.ret = inhaler retention. Ind. Port = induction port. *N* = 3.

A cascade impaction analysis was conducted to assess whether the cryomilled sa-mRNA–LNP powder possesses the required aerodynamic properties for deep lung deposition. The analysis demonstrated particle deposition across all stages of the cascade impactor (Figure 6). Inhaler retention was low, with 18.2 ± 3.3% of powder remaining in the device (Figure 6). The FPF of the emitted dose was 36.91 ± 2.73% and the average MMAD of the delivered dose was 4.13 ± 0.26 µm, aligning with the aerodynamic criteria for deep lung delivery (Table 2, Figure 6). However, a substantial amount of powder (28.1 ± 2.8%) accumulated in the induction port (Figure 6). This observation can likely be attributed to two factors: the relatively high powder dose loaded into the device (25 mg) and the occurrence of electrostatic charging under the low RH conditions (<30%) at which the experiment was conducted [49]. Electrostatic charging increases particle–surface interactions, leading to enhanced adhesion of particles to the walls of the induction port. This effect is further amplified by the geometry of the standard NGI induction port, which contains a 90° bend that does not accurately mimic the human oropharyngeal tract. It is anticipated that such deposition may be reduced in vivo, since the high humidity of the respiratory tract substantially lowers electrostatic interactions. Furthermore, the use of more anatomically realistic throat models, such as the Alberta Idealized Throat, may provide more accurate estimates of aerosol deposition in future studies [58]. Overall, these findings suggest that the cryomilled sa-mRNA–LNP formulation, containing sucrose and leucine, is well-suited for effective pulmonary deposition when administered via the Cyclops DPI.

The findings indicate that a dry powder formulation suitable for pulmonary administration can be achieved while maintaining the functional integrity of sa-mRNA–LNPs. Nevertheless, additional research is required to fully establish the therapeutic potential of these stabilized systems. In particular, future studies should assess pulmonary tolerability and determine the effective dose of sa-mRNA–LNPs in relevant animal models, alongside evaluation of mRNA expression and biological activity in vivo. These investigations represent a subsequent phase of development that follows successful technical optimization, which is the primary focus of the present study. Once this technological foundation is established, in vivo studies addressing bioavailability, pharmacokinetics, biodistribution, and therapeutic performance will be essential. Such work will enable definition of appropriate dosing strategies, delivery regimens, and safety profiles, thereby supporting the translation of sa-mRNA–LNP-based approaches toward clinical application.

## 4. Conclusions

The rapid deployment and efficacy of conventional mRNA–LNP vaccines during the COVID-19 pandemic underscored the potential of RNA-based platforms for infectious disease control. However, their widespread implementation is constrained by the need for ultra-cold storage and by the need for intramuscular administration, which is suboptimal for inducing mucosal immunity against respiratory pathogens. Overcoming these barriers requires formulations that combine enhanced stability with suitability for pulmonary delivery, thereby directly targeting the primary site of infection. sa-mRNA–LNPs represent a promising next-generation platform, offering the potential to achieve strong immune responses at lower doses compared to conventional mRNA–LNP vaccines. In this study, we showed that freeze-drying preserves the functionality of sa-mRNA–LNPs, even though it causes minor structural changes in the LNPs. The resulting freeze-dried powders remained stable at 4 °C for up to 8 weeks. Cryomilling in the presence of leucine produced respirable dry powders and retained approximately 60% of the original sa-mRNA–LNP activity. Importantly, this remaining functional fraction stayed stable during storage. The resulting dry powder formulation exhibited aerodynamic diameters within the optimal 1–5 µm range and was efficiently dispersed using the Cyclops DPI. Overall, the stabilization technology described in this paper holds the promise for the improved logistical flexibility for sa-mRNA–LNP vaccines, whereas the properties of the cryomilled powders make them suitable for pulmonary administration through inhalation.

## Figures and Tables

**Table 1:** Dispersion behavior from the Cyclops DPI of cryomilled freeze-dried sa-mRNA–LNPs with sucrose and 4 wt-% leucine.

Cyclops DPI	sa-mRNA–LNPs Cryomilled with 4 wt-% Leucine
X50 (µm)	3.04 ± 0.44
FPF (%)	69 ± 7
Retention (%)	17 ± 2
Inhaled dose (mg)	15.47 ± 2.28

FPF = fine particle fraction, fraction ≤ 5 µm. DPI = dry powder inhaler.

**Table 2:** Cascade impaction analysis of cryomilled freeze-dried sa-mRNA–LNPs with sucrose and 4 wt-% leucine.

Cyclops DPI	sa-mRNA–LNPs Cryomilled with 4 wt-% Leucine
Nominal dose (%)	29.65 ± 0.84
Emitted dose (%)	36.91 ± 2.73
Delivered dose (%)	58.42 ± 3.59
MMAD (µm)	4.13 ± 0.26

DPI = dry powder inhaler.

## Data Availability

Data are contained within the article or Supplementary Material. Further inquiries can be directed to the corresponding authors.

## Supplementary Materials

The following supporting information can be downloaded at: https://www.mdpi.com/article/10.3390/pharmaceutics18010121/s1, Figure S1: Viability of HeLa cells after adding sa-mRNA–LNPs; Figure S2: The glass transition (Tg) temperature of freeze-dried 20% sucrose in 20 mM HEPES buffer; Figure S3: Figure S3. DVS measurement of freeze-dried sucrose in HEPES buffer. Figure S4. Nanosight tracking analysis (NTA) of sa-mRNA–LNPs.

## Abbreviations

The following abbreviations are used in this manuscript:

DPI	Dry powder inhaler
DVS	Dynamic vapor sorption
DSC	Differential scanning calorimetry
FPF	Fine particle fraction
MEM	Modified Eagle’s Medium
MMAD	Mass median aerodynamic diameter
MOC	Micro-orifice collector
NGI	Next generation impactor
PBS	Phosphate-buffered saline
RH	Relative humidity
SD	Standard deviation
Tg	Glass transition temperature
