# Supplementary material for: Freeze-Drying in Sucrose Followed by Cryomilling Enables the Formulation of sa-mRNA–LNP Powders for Inhalation
Source: Pharmaceutics. 2026 Jan 18;18(1):121. doi: 10.3390/pharmaceutics18010121 (PMC12844638; doi:10.3390/pharmaceutics18010121)
Supplement: Supplementary file 1 [file pharmaceutics-18-00121-s001.zip › pharmaceutics-4062890-supplementary.pdf]

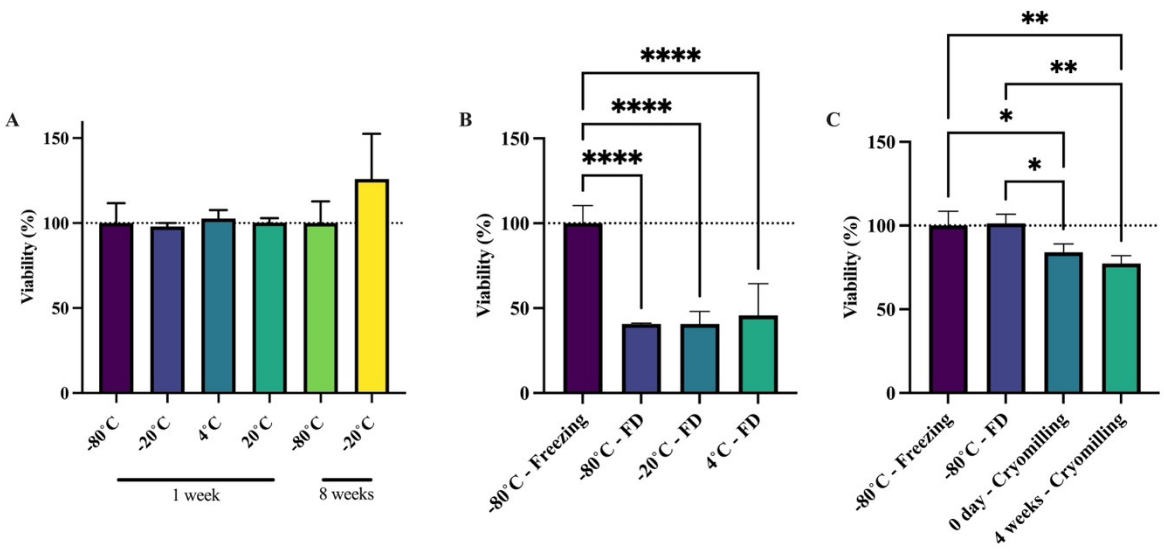

Figure S1. Viability of HeLa cells after adding sa-mRNA–LNP. A. Viability as percentage (%) of the -80 °C frozen sa-mRNA–LNP. The sa-mRNA–LNP were frozen in 20% sucrose and 20 mM HEPES buffer for 1 week and 8 weeks at −80 °C, −20 °C, 4 °C and 20 °C. B. Viability as percentage (%) of the -80 °C frozen sa-mRNA–LNP. The sa-mRNA–LNP were frozen at −80 °C with 20% sucrose and 20 mM HEPES buffer or freeze-dried in 20% sucrose and 20 mM HEPES buffer and then stored at −80 °C, −20 °C and 4 °C for up to 8 weeks. C. Viability as percentage (%) of the -80 °C frozen sa-mRNA–LNP. The sa-mRNA–LNP were frozen at −80 °C with 20% sucrose and 20 mM HEPES buffer or the sa-mRNA–LNP were freeze-dried with 20% sucrose and 20 mM HEPES buffer and stored at −80 °C or the sa-mRNA–LNP were cryomilled upon freeze-dried with 20% sucrose and 20 mM HEPES buffer. $N=3$. *$p < 0.05$, **$p < 0.01$.

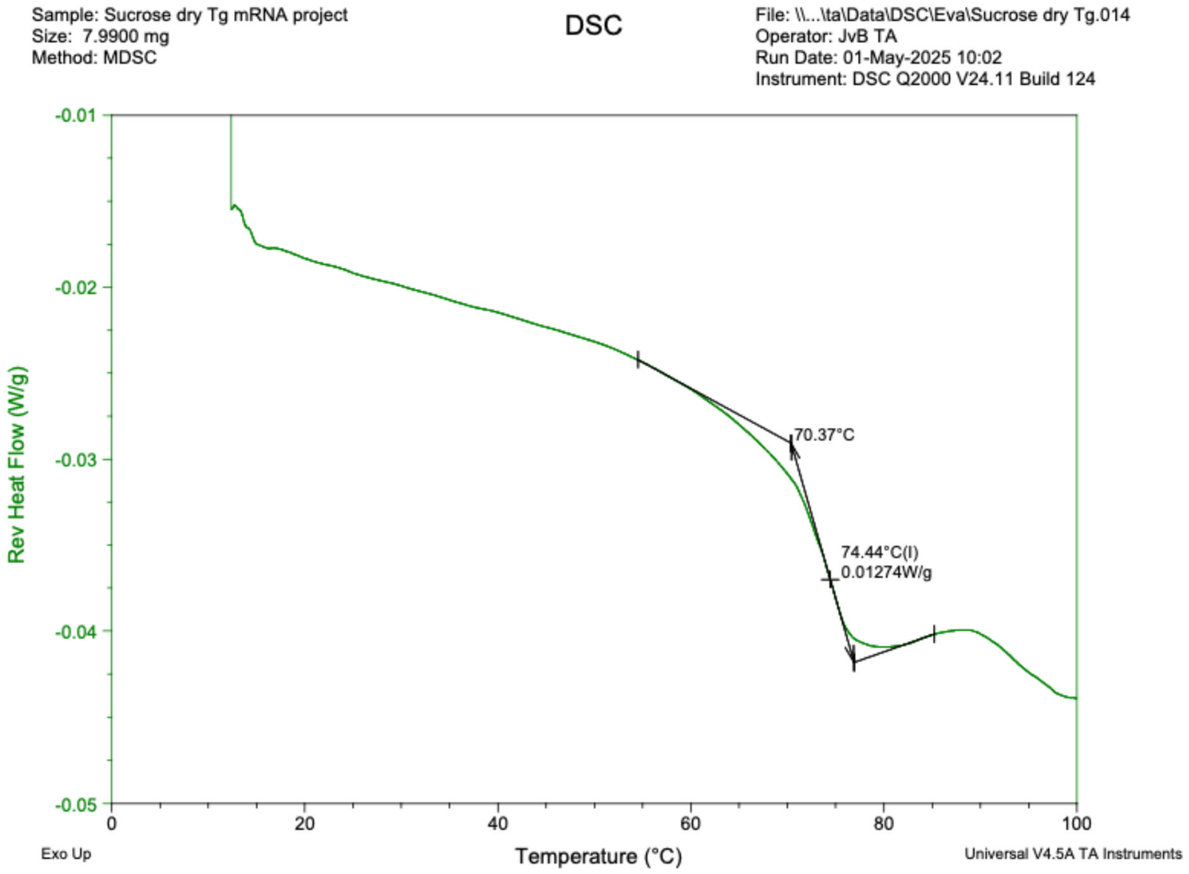

Figure S2. The glass transition (Tg) temperature of freeze-dried 20% sucrose in 20 mM HEPES buffer. A typical example of a triplicate measurement is shown. *N=3*.

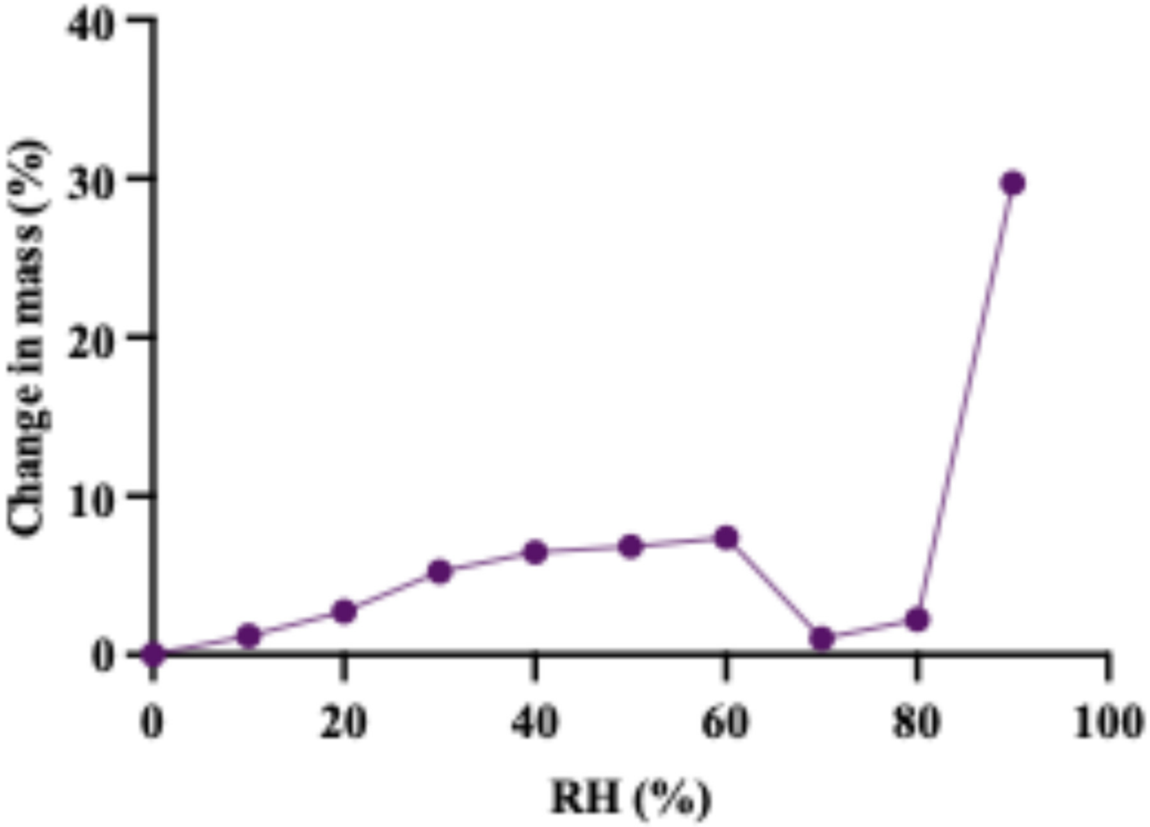

Figure S3. DVS measurement of freeze-dried sucrose in HEPES buffer. The change in mass and relative humidity (RH) is shown in %. *N=1*.

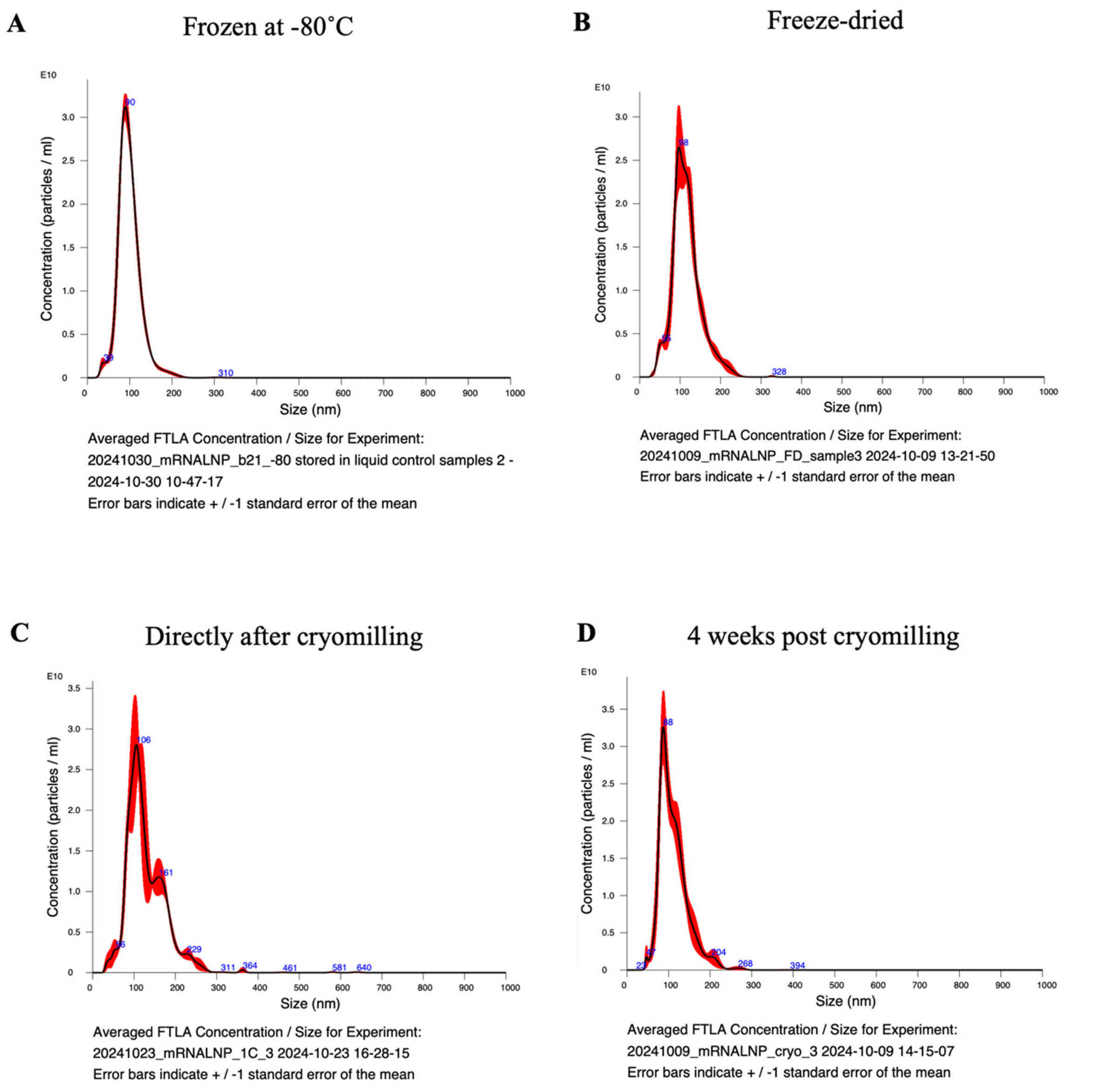

Figure S4. Nanosight tracking analysis (NTA) of sa-mRNA–LNP. A typical example of a triplicate measurement is shown. A. sa-mRNA–LNP supplemented with 20% sucrose and 20 mM HEPES buffer stored at −80 °C. B. Freeze-dried sa-mRNA–LNP supplemented with 20% sucrose and 20 mM HEPES buffer stored at −80 °C. C. Freeze-dried and cryomilled sa-mRNA–LNP supplemented with 20% sucrose and 20 mM HEPES buffer, measured directly after cryomilling. D. Freeze-dried and cryomilled sa-mRNA–LNP supplemented with 20% sucrose and 20 mM HEPES buffer stored for 4 weeks at −80 °C. *N=3*.
